# Identification of the significant pathways of Banxia Houpu decoction in the treatment of depression based on network pharmacology

**DOI:** 10.1371/journal.pone.0239843

**Published:** 2020-09-30

**Authors:** Zi-ying Chen, Dan-feng Xie, Zhi-yuan Liu, Yong-qi Zhong, Jing-yan Zeng, Zheng Chen, Xin-lin Chen

**Affiliations:** 1 Shenzhen Clinical College, Guangzhou University of Chinese Medicine, Guangzhou, China; 2 Department of Stomatology, The Third Affiliated Hospital of Sun Yat-sen University, Guangzhou, China; 3 School of Basic Medical Science, Guangzhou University of Chinese Medicine, Guangzhou, China; University of Colorado Denver Skaggs School of Pharmacy and Pharmaceutical Sciences, UNITED STATES

## Abstract

Banxia Houpu decoction (BXHPD) has been used to treat depression in clinical practice for centuries. However, the pharmacological mechanisms of BXHPD still remain unclear. Network Pharmacology (NP) approach was used to explore the potential molecular mechanisms of BXHPD in treating depression. Potential active compounds of BXHPD were obtained from the Traditional Chinese Medicine Systems Pharmacology Database and Analysis Platform Database. STRING database was used to build a interaction network between the active compounds and target genes associated with depression. The topological features of nodes were visualized and calculated. Significant pathways and biological functions were identified using Gene Ontology and Kyoto Encyclopedia of Genes and Genomes analyses. A total of 44 active compounds were obtained from BXHPD, and 121 potential target genes were considered to be therapeutically relevant. Pathway analysis indicated that MAPK signaling pathway, ErbB signaling pathway, HIF-1 signaling pathway and PI3K-Akt pathway were significant pathways in depression. They were mainly involved in promoting nerve growth and nutrition and alleviating neuroinflammatory conditions. The result provided some potential ways for modern medicine in the treatment of depression.

## Background

Depression is a widespread chronic mental illness characterized by low mood, sadness, and insomnia. Depression affects physical health [1]. The world health organization predicts that depression will be the leading cause of death of burden among all health diseases by 2030 [2].

Current therapies, including selective 5-serotonin reuptake inhibitors, serotonin-noradrenergic reuptake inhibitors and tricyclic antidepressants [3], can alleviate some major symptoms of depression. But these medications trigger a series of serious side effects like anxiety, gastrointestinal discomfort, drug resistance, withdrawal response and so on [4]. Therefore, it is necessary to develop safe, effective new drugs and therapies with lower side effects. Studies have shown that the use of complementary and alternative medicine (CAM) for the treatment of depression is common [5, 6]. As the important part of the CAM, traditional Chinese medicine (TCM) reported positive results for managing depression: less adverse reactions than other antidepressants and no significant differences from medication [7, 8].

Banxia Houpu decoction (BXHPD) is a famous Chinese medicine prescription, which firstly recorded in Jingui Yaolue (Synopsis of Prescriptions of the Golden Chamber) in Eastern Han Dynasty of Chinese history (25AD–220AD). BXHPD was 5 herbs, which included Pinellia rhizome (PR, Banxia), Magnolia officinalis cortex (MOC, Houpu), Poria (PO, Fuling), ginger rhizome (GR, Shengjiang), and Perilla folium (PF, Zisu). In the theory of TCM, BXHPD could promote chi, eliminate stagnation, calm the adverse chi and dissolve phlegm. BXHPD was widely used in the treatment of depression and achieved effective results [9, 10]. Zheng Q et al reported that the effective rate of BXHPD combined with western medicine was 94.5%, and the cure rate was 55.2%, compared to 81.1% and 22.9% treated with western medicine alone in the treatment of depression [10].

Some previous studies predicted the molecular mechanism on depression of BXHPD, which mainly included activation of the inflammatory response system [11], monoamine hypothesis [12], Hypothalamic Pituitary Adrenal (HPA) axis dysfunction [13], low expression of brain-derived neurotrophic factor (BDNF) [14] and so on. The biological mechanism in holistic manner are still unknown. The main problems are as follows: (1) On account of the complex composition, the biological effects on depression of BXHPD were not unified. BXHPD included many active compounds, most of which were different in therapeutic mechanism. For instance, Baicalein [15] and Luteolin [16] were related to the suppression of inflammation about nerve through the regulation of pathways or the expression level of inflammatory related factors. Beta-sitosterol might affect the content of neurotransmitter [17]. (2) The previous studies mainly focused on the gene targets or biological functions rather than multiple pathways: Anti-depression on BXHPD proved to elevate brain 5-hydroxytryptamine (5-HT) levels, attenuate abnormalities in dopaminergic system functions [18], ameliorate the damages of lipid peroxidation [19], and adjust the amino acid metabolism and energy metabolism [20].

**Fig 1:**
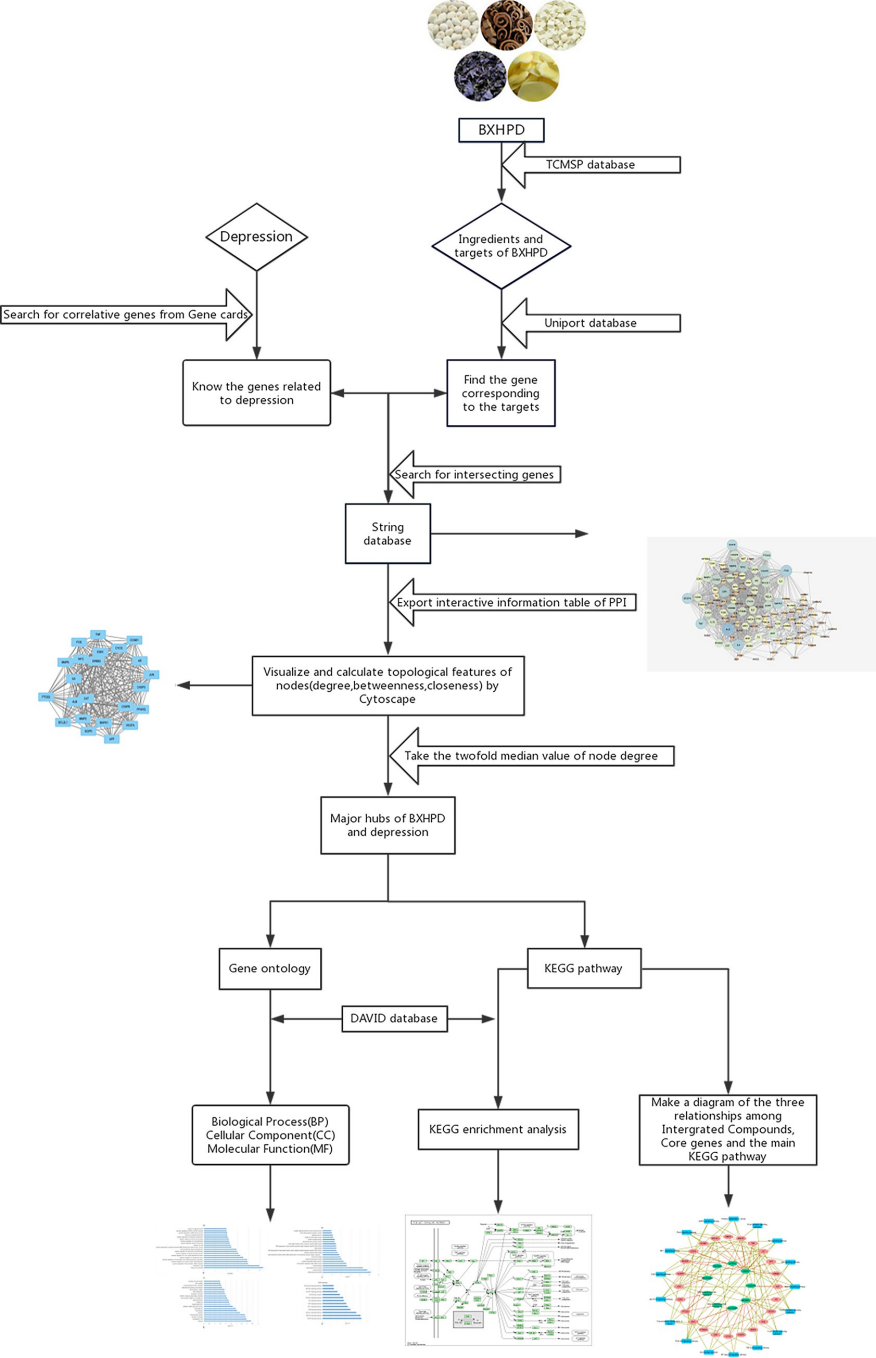
The workflow of the experimental procedures.

In order to explore details of the mechanism and relevant pathways, it is essential to elucidate the molecular and biological basis of TCM preparations and NP approaches have been proven to be a powerful approach [21, 22]. The holistic philosophy of TCM shares similar characteristic of NP. TCM network pharmacology approach was established by "network target, multi-components" mode, which predicted the target profiles, revealed drug-gene-disease co-module associations, and interpreted the combinatorial rules and network regulation effects of herbal formulae [23]. Herein we focused on the following issues: (1) Which active ingredients were involved in the regulation of depression? (2) Which active ingredients and proteins regulated the target to achieve the biological activity? (3) What pathways or biological processes did the active ingredients regulate? The main contribution of our work was to clarify the potential mechanism of BXHPD in the treatment of depression using NP. The workflow of the experimental procedures was showed in Fig 1.

## Methods

### Identification of active compounds

The main chemical constituents of BXHPD were collected from the Traditional Chinese Medicine Systems Pharmacology Database and Analysis Platform Database (TCMSP) (http://tcmspw.com/tcmsp.php/) [24]. TCMSP database was a network pharmacological database of Chinese herbal medicines and it provided pharmacokinetic information for each compound [25], such as drug similarity (DL) and oral bioavailability (OB). Compounds with good drug-like were selected according to their characteristics (including absorption, distribution, metabolism and excretion [ADME]) [25].

OB and DL were important indicators to evaluate ADME [26] characteristics. OB represented the correlation of effective compounds. OB ≥30% meant that the effective compounds had a strong correlation. DL of new molecules was assessed using the following formula [27]: $f(A,B)=\frac{A\cdot B}{{\left|A\right|}^{2}+{\left|B\right|}^{2}-A\cdot B}$. "A" is the molecular descriptor of BXHPD based on Dragon software (http:www.talete.mi.it/products/dragon_description.htm). "B" represents the average descriptor of all drugs based on The Drug Bank database [28]. The Dragon software calculates the average of all descriptors and the active molecules with DL ≥ 0.18 were selected. Therefore, compounds with OB ≥ 30% and DL ≥ 0.18 were selected for further study. Pinellia ternata, Magnolia officinalis, Poria cocos, Ginger and Perilla frutescens were used separately as search terms to gain the pharmacokinetic information of active compounds.

### Screening of potential targets

An important step after screening active compounds was to identify their molecular targets for triggering biological effects [29]. UniProt (https://www.uniprot.org/) is a protein database with abundant information and extensive resources [30]. The protein targets retrieved from TCMSP database were put into Uniprot, and the corresponding gene names were extracted from UniProt KB. We restricted species to human beings and converted them into official gene names. Gene Cards (https://www.genecards.org/) is a searchable and comprehensive database which automatically integrates data from about 125 web-based sources of genes, including genomes, transcriptomes and proteomics [31]. Gene Cards database is applied to search disease targets for depression. Potential antidepressant targets of BXHPD were obtained by intersecting the targets of active ingredients and potential disease-related targets.

### Construction and topological analysis of PPI network

STRING (https://string-db.org/cgi/input.pl) was used for expanding protein-protein interaction (PPI) network data [32]. The gene names of potential targets converted from UniProt were put into STRING to download interactive information table of PPI. Cytoscape V3.7.1 was used to visualize PPI and complete topological analysis [33]. Core genes of BXHPD for anti-depression were obtained meeting degree ≥ 38 (two times of median of all nodes).

"Degree", "Betweenness" and "Closeness" were used to assess the topological importance of the selected core genes [34]. "Degrees" is defined as the number of links to a node that reflects the node's interaction with another node. Nodes with an extremely high level of degree tend to be critical in interaction networks. "Betweenness" is defined as the number of closest associations. "Closeness" indicates the sum of the nodes distance from all other nodes.

### Biological function enrichment and metabolic pathway acquisition

The Gene Ontology (GO) is a database that functionally marks genes and proteins as three main terms: biological process (BP), cellular component (CC) and molecular function (MF) [35]. The function of genes can be defined and described in many ways through CC, BP and MF [36]. Kyoto Encyclopedia of Genes and Genomes (KEGG) database is used to determine advanced functions and biological correlation of a large number of genes [37]. KEGG pathways infer the relationships between proteins and the biological functional annotation of proteins. David 6.8 is used for GO and KEGG pathway analysis of the core antidepressant targets of BXHPD [38]. The corresponding data were obtained by using human genes as the range and *P* ≤ 0.05 as the screening value. The relevant biological processes or related pathways with the largest number of corresponding target points were selected to draw bar charts.

### Construction of a compound-target-pathway network

The active compounds, core targets and signal pathways were utilized to construct a compound-target-pathway (C-T-P) network. In this C-T-P network, "nodes" of different colors represented the protein targets, compounds, signal pathways or diseases. "Edge" indicated the interaction of compound targets, compound pathways, or diseases. The networks were constructed by using Cytoscape v3.7.1.

## Results

### Active compounds of BXHPD

In the TCMSP database, 13 kinds of compounds from PR, 15 from PO, 5 from GR and 14 from MOC were included. After removed 5 duplicates from 49 compounds, 44 compounds were further studied (Table 1).

**Table 1:** Basic information of active compounds of BXHPD.

MOL ID	MOL NAME	OB%	DL	TARGETS NUMBER
MOL000006	luteolin	36.16	0.25	55
MOL002714	baicalein	33.52	0.21	34
MOL000358	beta-sitosterol	36.91	0.75	34
MOL000449	Stigmasterol	43.83	0.76	29
MOL002670	Cavidine	35.64	0.81	26
MOL005970	Eucalyptol	60.62	0.32	24
MOL002773	beta-carotene	37.18	0.58	23
MOL000296	hederagenin	36.91	0.75	22
MOL000519	coniferin	31.11	0.32	21
MOL000492	(+)-catechin	54.83	0.24	7
MOL001749	ZINC03860434	43.59	0.35	4
MOL005980	Neohesperidin	57.44	0.27	4
MOL006957	(3S,6S)-3-(benzyl)-6-(4-hydroxybenzyl)piperazine-2,5-quinone	46.89	0.27	3
MOL006129	6-methylgingediacetate2	48.73	0.32	3
MOL000953	CLR	37.87	0.68	3
MOL001755	24-Ethylcholest-4-en-3-one	36.08	0.76	2
MOL005030	gondoic acid	30.7	0.2	2
MOL006936	10,13-eicosadienoic	39.99	0.2	2
MOL003578	Cycloartenol	38.69	0.78	2
MOL006967	beta-D-Ribofuranoside, xanthine-9	44.72	0.21	2

In the 44 compounds, 12 compounds had no protein targets. A total of 159 protein targets for 32 compounds were obtained from TCMSP. After inputting the 159 protein targets into the Uniprot, we found that 11 protein targets could not found the corresponding genes. The 11 protein targets above were removed. Adding up to 148 gene targets and 32 compounds were preserved for further researches.

### The construction of compounds-target network

**Fig 2:**
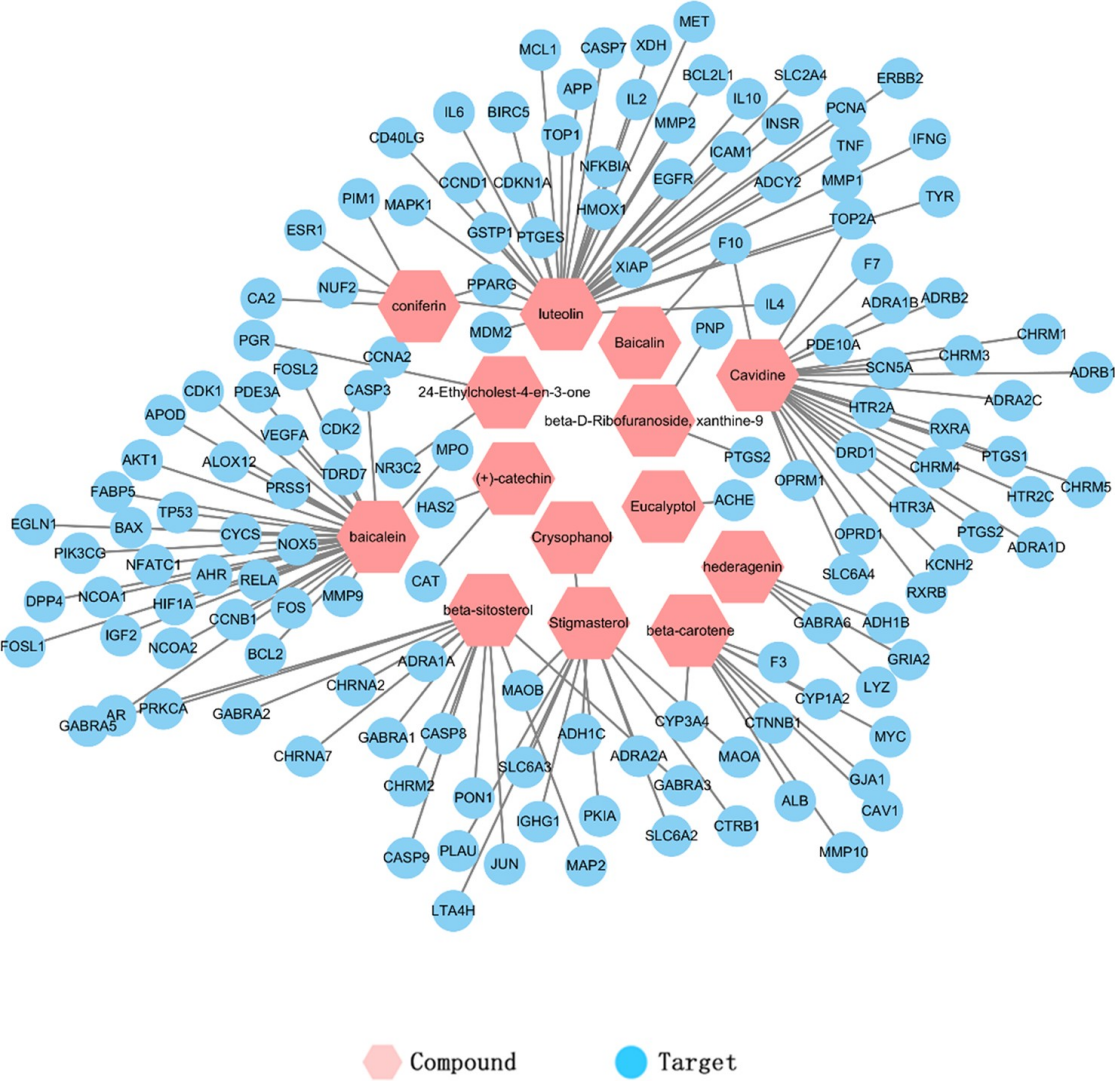
Compounds-target network. Pink hexagons represent compounds and blue circles represent targets.

In order to identify the relationship between TCM and the corresponding targets [39], a compounds-target network was built to show the correspondence of active BXHPD compounds and targets (Fig 2). The compounds with the most amount of targets contained luteolin (55), baicalein (34), beta-sitosterol (34) and stigmasterol (29).

### Acquisition of core targets

**Fig 3:**
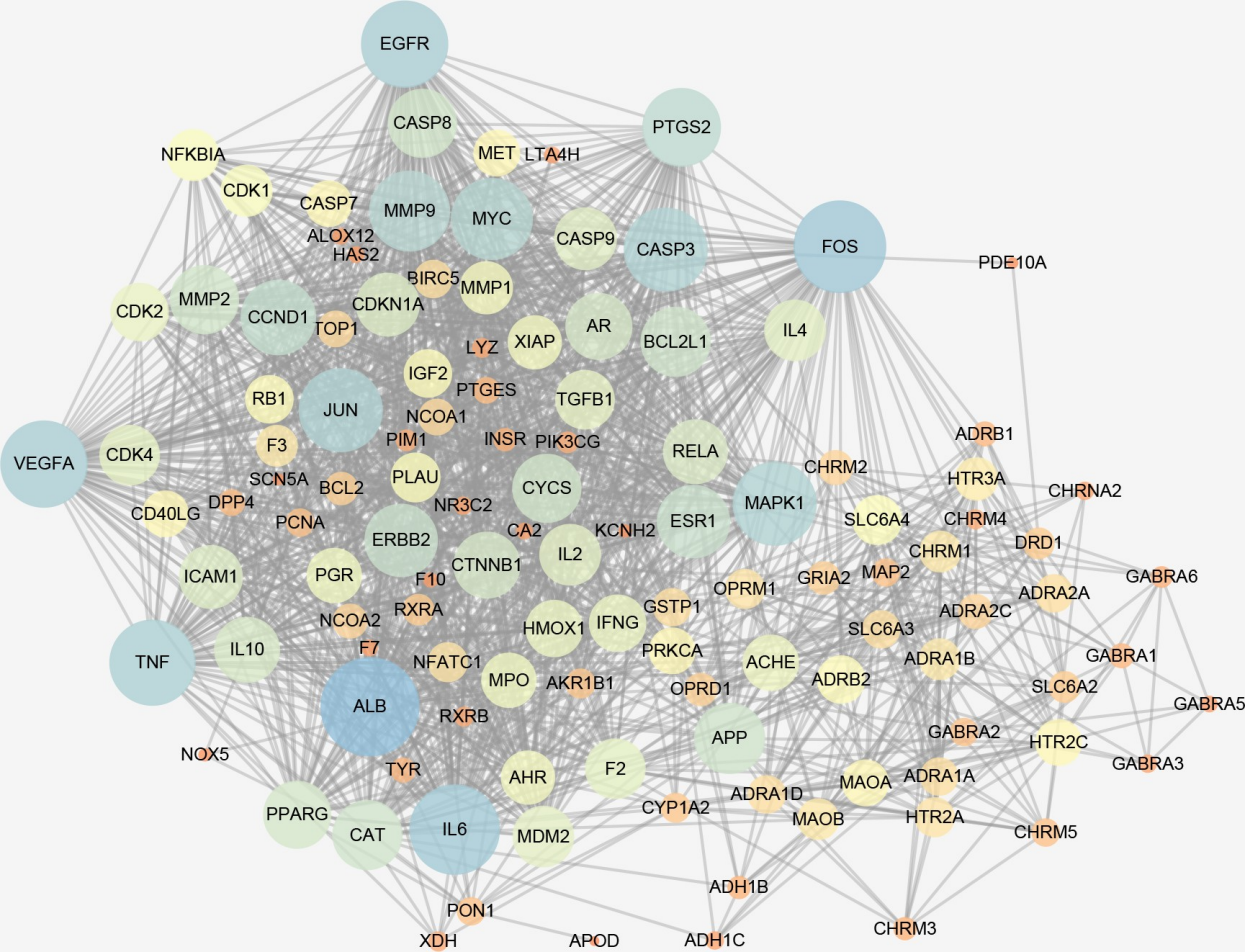
PPI network.

From Gene Cards database, 8,959 genes were related to depression. After intersecting 148 potential active targets with 8,959 depression related genes, 121 potential drug target genes were obtained. The PPI network was built by STRING (Fig 3). The PPI network consisted of 120 nodes and 1476 edges. After the visual topological analysis by CytoNCA (an application in Cytoscape), 24 core targets were obtained (Table 2). Among the 24 core targets, ALB has the greatest degree (71), followed by FOS (65), IL6 (63) and EGFR (60).

**Table 2:** Core targets of BXHPD.

Gene symbol	Degree	Gene symbol	Degree
ALB	71	CCND1	49
FOS	65	ERBB2	47
IL6	63	ESR1	47
EGFR	60	APP	45
VEGFA	60	CAT	44
TNF	59	BCL2L1	44
MAPK1	57	MMP2	43
JUN	57	CYCS	43
CASP3	57	CASP8	43
MYC	55	PPARG	42
MMP9	54	CTNNB1	41
PTGS2	52	AR	41

### GO and KEGG pathways analysis for core genes

**Fig 4:**
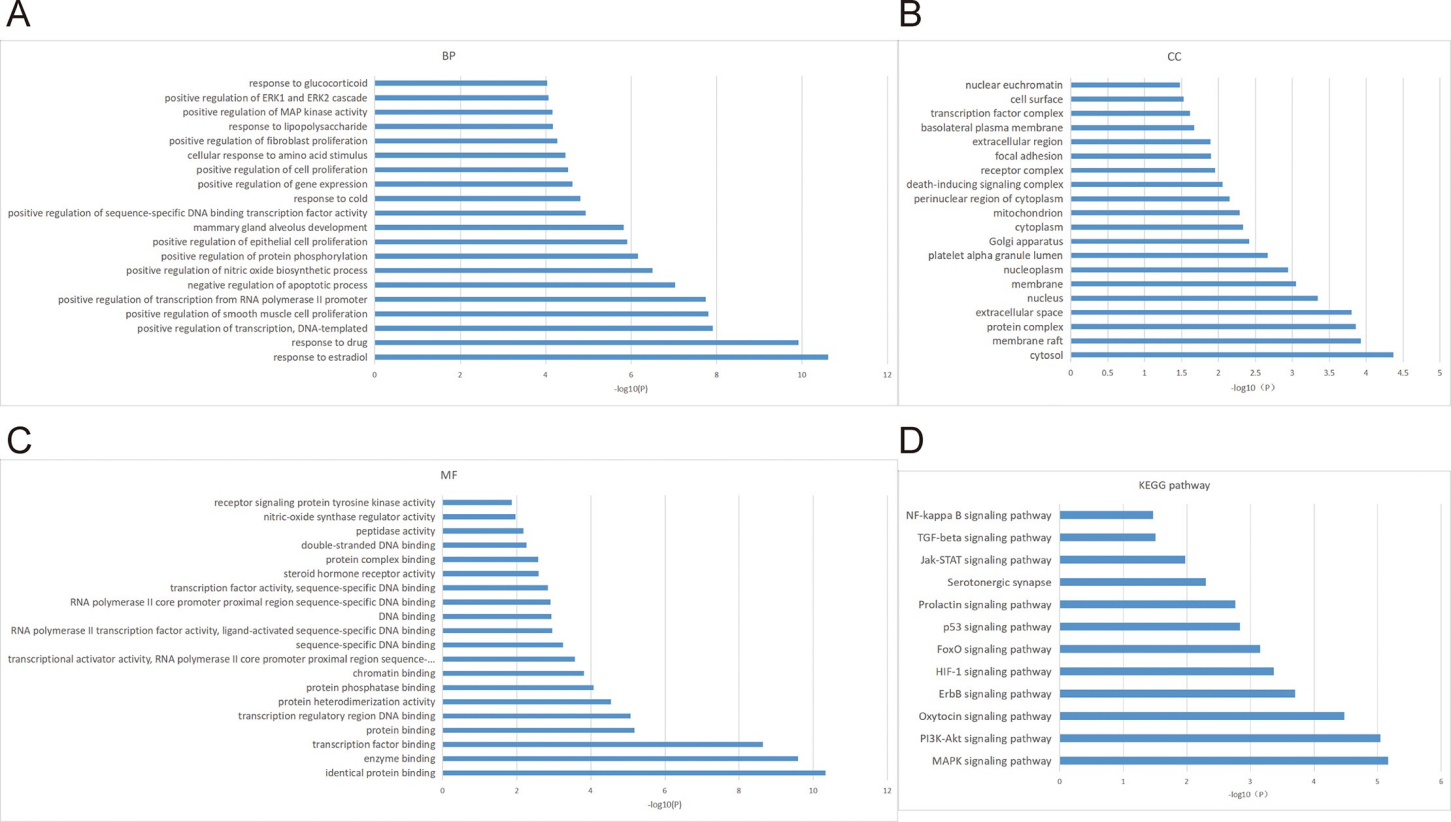
GO and KEGG functional analysis. (A) GO biological process terms. (B) GO cellular component terms. (C) GO molecular function terms. (D) KEGG pathway.

GO enrichment analysis and KEGG pathways analysis were performed on the 23 target genes by David (Fig 4). Pathways with P-value<0.05 were considered as significant pathways.

GO enrichment analysis contains three broad categories: BP, CC and MF. BP terms mainly contained response to estradiol, response to drug, positive regulation of transcription, DNA-templated, positive regulation of smooth muscle cell proliferation and positive regulation of transcription from RNA polymerase II promoter. CC terms mainly contained cytosol, membrane raft, protein complex, and extracellular space and nucleus. MF terms mainly contained identical protein binding, enzyme binding, transcription factor binding and protein binding. KEGG pathways mainly contained MAPK signaling pathway, PI3K-Akt signaling pathway, Oxytocin signaling pathway, ErbB signaling pathway and HIF-1 signaling pathway.

### KEGG enrichment analysis

**Fig 5:**
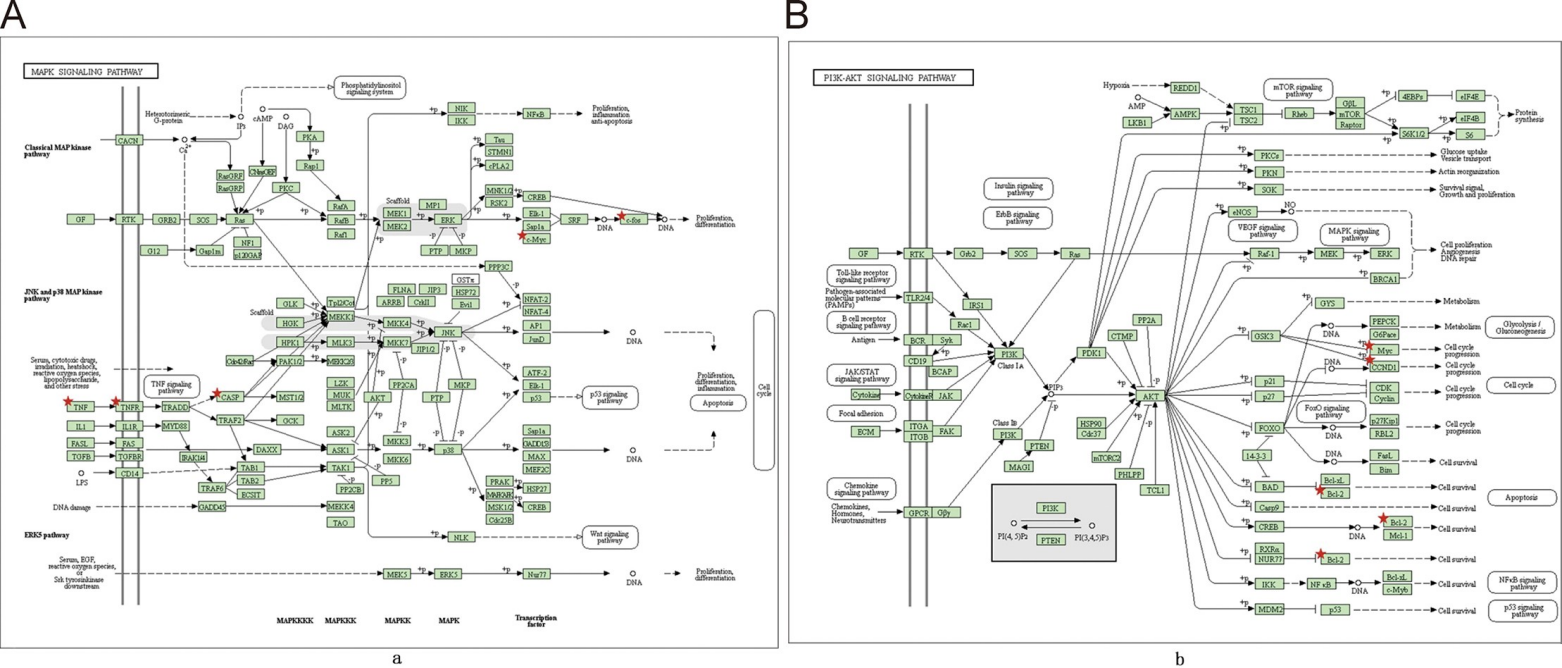
KEGG enrichment diagram. (A) MAPK signaling pathway. (B) PI3K-AKT signaling pathway. Red asterisks represent core targets.

KEGG Enrichment Analysis reveals the possible biological processes of pathway by analyzing biological processes, which unlocks the molecular roles of targets in the treatment of depression. There were more than 20 pathways associated with depression. In these pathways, MAPK signaling pathway (Fig 5A) and PI3K-Akt signaling pathway (Fig 5B) were most closely related to depression.

One of the MAPK signaling pathway: ERK, part of the MAPK pathway in the prefrontal cortex and hippocampal played an important role in antidepressant processing [40]. ERK activates Elk-1, Sapla and c-Myc through phosphorylation. Elk-1, Sapla and c-Myc combine to form SRF, SRF activates c-fos through DNA, c-fos produces proliferation differentiation function, via DNA activation. The PI3K-Akt signaling pathway was one of the important pathways of depression. GF activates RTK on cell membrane, RTK activates IRS1, IRS1 activates PI3K(Class IA), PI3K activates AKT through PIP3, AKT inhibits BAD by phosphorylation, BAD ultimately affects Cell survival by inhibiting Bcl-xL and Bcl-2.

### Component-target-pathway network

**Fig 6:**
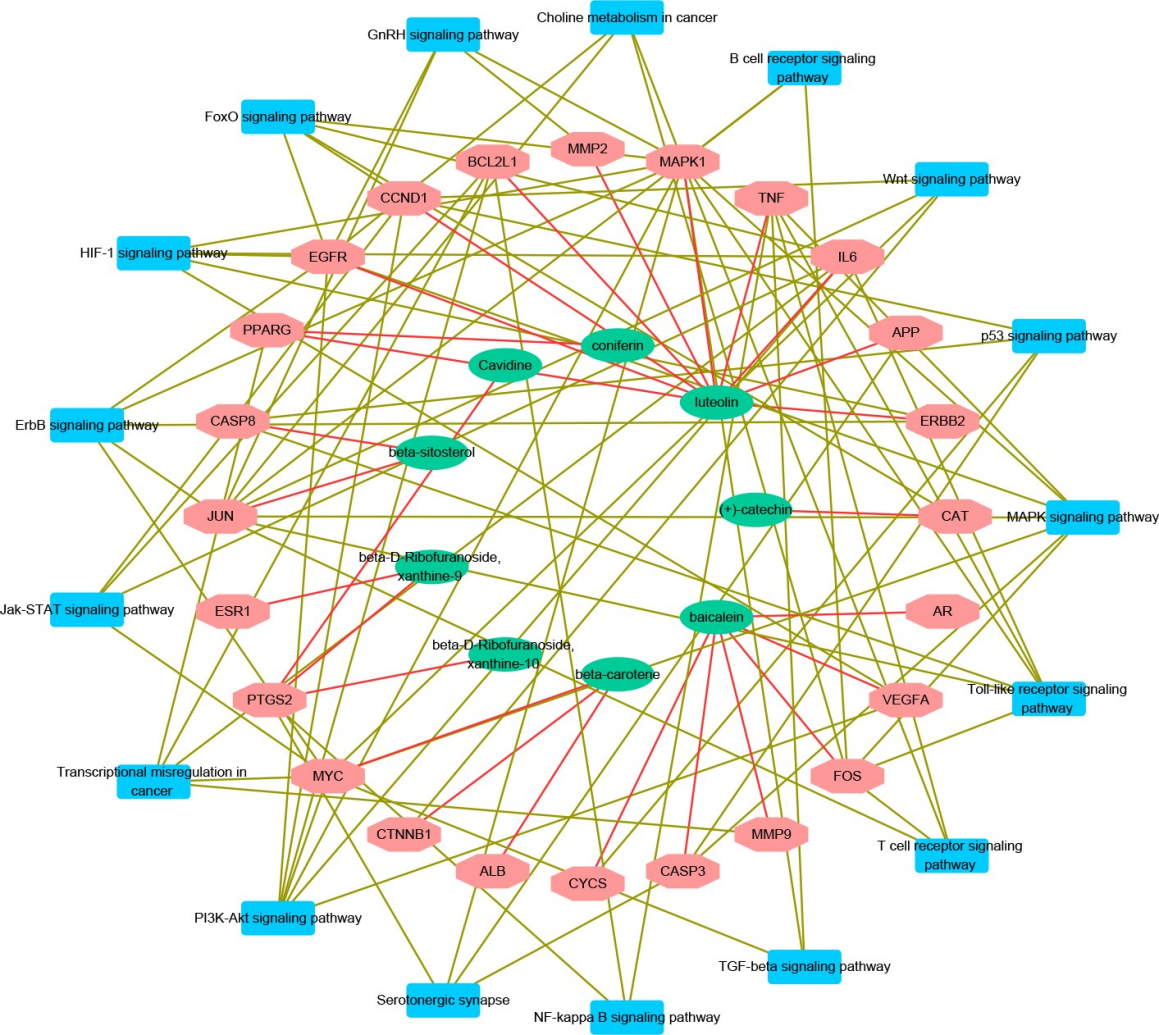
Compound-target-pathway networks. Green nodes stand for chemical compounds in BXHPD; Red nodes stand for known target genes from chemical compounds and depression; Blue nodes stand for the main biological pathway; Red lines stand for the relationship between compounds and target genes; Brown lines stand for the relationship between target genes and biological pathway.

Compound-Target-Pathway networks of BXHPD were shown in Fig 6. There were 9 compounds, 24 targets and 17 pathways. The nodes represent drug compounds, active components and targets. The edges represent their relationship. Through the network, we could find that some pathways had high enrichment in the network such as PI3k-Akt pathway, which suggested that these pathways may play a key role in therapeutic effects of BXHPD.

## Discussion

Through the screening of active compounds, Luteolin (53 targets), Baicalein (34 targets), Beta-sitosterol (34 targets), Stigmasterol (29 targets) and Cavidine (26 targets) might have primary effects for antidepression in BXHPD. (1) As an active flavonoid derived from astragalus root, baicalein could alleviate the development of depressive symptoms by up-regulating the level of dopamine in the hippocampus and the level of BDNF [41]. Additionally, it stimulated the activity of Erks, a member of MAPK family [42], and inhibited the down-regulation of nuclear factor-kappa B (NF-κB) pathway [43, 44], such as IL-6 and TNF-α, to inhibit neuroinflammation and to protect nerves. (2) Luteolin was also a flavonoid from a variety of plants that prevented nerve cell death [45] and suppressed the levels of inflammatory factors such as IL-6 and TNF-α to reduce nerve damage [46]. Not only the activity of Acetylcholinesterase and antioxidant enzymes could be improved, but the formation of nitric oxide could be done by luteolin to achieve antioxidant and anti-inflammatory effects [16]. (3) Cavidine was an isoquinoline alkaloid from Corydalis impatiens, which was an anti-inflammatory substance that inhibited NF-κB pathway related pro-inflammatory substances such as IL-6 and TNF-α [47, 48]. Cavidine might be related to the suppression of inflammation about nerve. (4) Zhao et al. found that beta-sitosterol affected the content of dopamine and 5-HT, which might be a potential drug ingredient target [49]. (5) Stigmasterol was famous for the treatment of depression in TCM, which might regulate the level of nerve steroids to control depressive symptoms [50].

Our results showed that the important pathways of BXHPD mainly contained PI3k-Akt signaling pathway, MAPK signaling pathway, Oxytocin signaling pathway, ErbB signaling pathway and HIF-1 signaling pathway. These pathways are linked to the core genes chiefly including FOS, IL6, TNF-α, Bcl-2, c-Jun, ERK, and EGF gene in our research in the treatment of depression. Those results were supported by lots of studies. (1) PI3k-Akt pathway phosphorylates the downstream factor Forkhead box O3a and inhibits neurotoxic apoptosis conducted by Corticosterone (CORT), which is the effective antidepressant process [51, 52]. In addition, PI3k-Akt pathway controlled BDNF (such as IL-6, TNF-α) to reverse depression-like behavior in a neurotrophic and neuroprotective way [53, 54]. PI3k-Akt pathway was related to neuroplasticity and could achieve anti-depression effect by enhancing the formation of synapses and the extension of axon dendrites [55, 56]. PI3k-Akt pathway also adjusted downstream molecular to achieve antidepressant effects, such as Bcl-2 [57] and c-Jun [58], both of which appeared in core targets of our research (Table 2). (2) MAPK pathway was a well-known pathway related to neuronal proliferation and differentiation among depression [59]. ERK, an important gene of MAPK pathway in the prefrontal cortex and hippocampal played an important role in antidepressant processing, such as increasing the expression of BDNF [40] and promoting growth-related microtubulin in the hippocampus [60]. Patel et al. showed that MAPK pathway could promote the formation of nitric oxide (NO), thus improving neuroplasticity and inhibiting apoptosis (Fig 4A) [61]. (3) Oxytocin signaling pathway affected nerve excitation transmission [62] through the modulation of 5-HT [63] and interaction with gamma-aminobutyric acid [64]. Wang et al. found that Oxytocin signaling pathway could reverse depression by down-regulating CORT and by affecting the HPA axis [65]. Oxytocin improved depression symptom by down-regulating c-fos protein and by inhibiting the ERK pathway as well [66]. (4) The ErbB pathway was also associated with depression by adjusting neuregulin and affecting downstream Akt and ERK signaling pathways [67, 68]. EGF and VEGF were parts of the ErbB family and were associated with nerve growth and nutrition [69]. (5) HIF-1 signaling pathway was also relevant to depression in our study. In the pathway, Hypoxia-inducible factor (HIF-1) was a main regulator in hypoxia response and played a role in energy supply in depression through neurotransmitter transmission [50]. (6) Other pathways in our result (Fig 4B) were indirectly linked to depression, in terms of nerve growth and inflammation, such as FoxO signaling pathway [70, 71], Jak-STAT signaling pathway [72, 73], prolactin signaling pathway [74, 75] and TGF-beta signaling pathway [76, 77].

Our research had some limitations. First, it was uncertain whether Chinese medicine was absorbed in the intestinal tract as the active compounds in our research. Second, core genes of specific pathways related to downstream products were not objectively presented in our results. Third, whether the role of pathways in our study was up-regulated or down-regulated was unclear. Fourth, our study used the network construction method to speculate the potential anti-depression targets of BXHPD, which still needs to be further verified by biological experiments.

## Conclusions

BXHPD can effectively alleviate the symptoms of depression through the molecular mechanisms predicted by NP. NP analysis demonstrates that there were multi-scale curative activity in regulating depression related biological processes. Pathway enrichment analysis indicated that MAPK signaling pathway, ErbB pathway, HIF-1 pathway and PI3K-Akt pathway were significant pathways in depression. They mainly were involved in promoting nerve growth and nutrition and alleviating neuroinflammatory conditions. This study provided some potential ways for modern medicine in the treatment of depression.

## Supporting information

S1 File (RAR)
